# Mechatronics Design of a Clinostat Agriculture Space System for Biomimetic Phyto-Growth in Microgravity (Phyto-G) and 3D-Motion Computer Simulation on Hydroponic Environment

**DOI:** 10.3390/biomimetics11050340

**Published:** 2026-05-14

**Authors:** Ricardo Barreto, Jose Cornejo, Mariela Vargas, Nicolas Gastello, Anghello Rodriguez

**Affiliations:** 1 Biomimetic Engineering and Aerospace Mechatronics (BEAM) Laboratory, Betta International Corporation, Lima 15026, Peru; doctor.engineer@ieee.org (J.C.);; 2 Betta Aerospace, Betta International Corporation, Lima 15026, Peru; 3 National University of Engineering, Lima 15333, Peru; 4 Betta College of Science and Technology (Betta-Tech), Betta International Corporation, Lima 15026, Peru; 5 Higher Technical School of Engineering and Industrial Design, Polytechnic University of Madrid, 28012 Madrid, Spain; 6 Universidad Tecnológica del Perú, Lima 15046, Peru

**Keywords:** biomimetic engineering, microgravity, mechatronics, agriculture, clinostat

## Abstract

So far, space exploration has attracted increasing scientific interest due to the growth of missions promoted by private investment, such as SpaceX, Boeing, Blue Origin, and the recent attention generated by astronomical phenomena such as 3I/ATLAS. However, access to space experimentation remains limited and expensive. For this reason, new approaches to simulate space conditions on Earth are being developed to broaden research opportunities bio-inspired by plant responses to phototropism and geotropism. In this context, Betta Aerospace has continued the development of a microgravity simulation system consisting of a 3-axis clinostat powered by a single motor, continuous external electrical supply, and, in this project, a continuous external liquid supply. The proposed pioneer system was designed as a flexible platform manufactured through reinforced 3D printing, with an approximate size of 30 cm, an estimated payload of 30 kg, and a 24 V supply. Its main goal is to study the effects of simulated microgravity on aquatic organisms while enabling longer observation times in a controlled freshwater environment. Candidate biological samples include *Ulva lactuca*, *Pyropia*, *Spirulina*/*Arthrospira*, and *Chlorella*. Preliminary motion tests confirmed continuous operation at 10 rpm. In addition, a simplified static finite element analysis under a 294 N load yielded a maximum von Mises stress of 5.45 × 10^7^ Pa and a maximum displacement of 1.73 mm.

## 1. Introduction

The year 2025 brought new achievements in space exploration, not only being funded by government agencies but also by private companies supporting the advance [1,2]. This fact has raised the interest of scientists in new fields or niche ones that would not be found by the budget cut due to state priorities. In human evolution, the shift from a nomadic to a sedentary lifestyle [3] was observed thanks to the mastery of controlling the environment to facilitate obtaining resources for life [4]. This began with observation and experimentation, ranging from simply sowing a seed in a field to having total and automated control of plant growth using only essential resources and optimizing them to the maximum [5], to now understanding deeper phenomena like the gravity effect and sunshine to directly impact plant growth, health, and efficiency.

In this context, to maximize productivity per unit area and resource efficiency, Controlled Environment Agriculture (CEA) [6,7] offers a technological framework for cultivating organisms under strictly regulated conditions, usually controlling lighting [8] (e.g., LEDs and photoperiod), nutrient delivery [9] (hydroponics or related systems), temperature [10], and atmospheric composition [11]. Since food, oxygen production, and carbon dioxide removal must be integrated into small, closely watched systems, often with automation and sensor feedback, CEA concepts are immediately applicable to space living [12,13]. Because they may be cultivated in aqueous bioreactors that facilitate high-density cultivation and continuous monitoring (pH, dissolved oxygen, optical density), algae and microalgae are excellent candidates within this paradigm, allowing for both nutritional biomass production and life-support activities [12,13]. Likewise, from the first moon landing to the continuous rotation of scientific personnel on space stations, space life has become increasingly sophisticated, to the point of being almost routine [14]. While all sources are managed and processed, food production remains limited by what is periodically sent to space [15,16]. Therefore, something as basic as a plant-based food source could remove the limitation on the capacity for longer or more distant missions [17].

Changing the physical surroundings might cause quantifiable physiological changes [18] in addition to logistical ones. Through modifications in cell wall remodeling, stress-related pathways, and hormone distribution (including auxin signaling), spaceflight and altered-gravity conditions can affect growth patterns and disturb gravitropism in higher plants [19,20,21]. The significance of controlled experimental platforms and sufficient controls is shown by transcriptomic meta-analyses across several spaceflight datasets that reveal conserved changes in gene expression associated with adaptation and stress responses [21]. Changes in productivity, pigment composition, antioxidant activity, and membrane lipid remodeling in photosynthetic microorganisms like microalgae can reflect changes in gravity and reactor hydrodynamics, offering measurable endpoints for controlled experiments and bio-automation systems [13,22]. Currently, both on Earth and in space, studies of living organisms such as very small plants [23] or animals [24] are limited due to logistical constraints and the risks of working with living matter in an environment isolated from any support [25]. It is impossible to fully recreate the space environment on Earth’s surface, even though certain mechanisms exist that can simulate microgravity [26,27,28]. These mechanisms are limited by simulation time, as well as the size and quantity of the samples.

Among the plethora of edible plant species, only a few compensate for their nutritional value with their maintenance [29,30,31]. For instance, the time between planting and harvest should not be too long, the size of the fully grown plant has to be minimal, the water must be streamlined among all travel needs, and the edible parts have to contribute to the crew’s diet [32,33]. On the other hand, when the first colonies arrive and settle on the planet, the new aim is to use extraterrestrial soil for growing plants in order to expand the plants’ source [34,35]. However, with the growth in interest in space exploration and the increase in private investment, the research field has widened to look for experiments for understanding life outside the Earth, such as the effect of microgravity on water-living beings like algae [36,37] and fish [38,39]. Although the investment has increased, the places for experimentation in a real microgravity environment are limited [40]. Moreover, gravity is a phenomenon not yet completely understood; instead, it is only calculated or simulated but not artificially created or controlled [41,42]. For those reasons, some sophistry machines are made in order to recreate a custom gravity strength [43,44,45]. In this case, one that could recreate a microgravity environment through continuous spin is the clinostat [46], which is able to increase the microgravity simulation by adding more spin frames. However, those frames could not interact with the inside of the machine, where the sample is located; the sample is only be able to take data using wireless devices, but not for refilling resources for maintenance [47].

This project aims to contribute to the growing interest in and need for solving the problems of a real microgravity environment by optimizing the design of a 3D clinostat, rather than waiting or investing large sums of money to send samples into space. It focuses on new hypotheses and seeks to pioneer the simulation of microgravity in an aqueous environment, using algae (*Ulva lactuca* [48] and *Pyropia* [49]) and *microalgae* (*cyanobacteria Spirulina* [50] and *Arthrospira* [51] or microalgae *Chlorella* [52]) as samples. This is achieved by assembling a domestic-sized aquarium instead of small containers or micro-containers with all the necessary environmental controls within a clinostat. The innovative design, based on the reviewed literature, allows for extended testing periods without needing to shut down the device. This uninterrupted plumbing and electrical system is located on the frame. In addition to the fact that movement stress could be a hardened stress, since they do not share the need to root in a substrate like aquatic plants [53], these algae have sufficient nutritional value to be used in space as food or a supplement for astronauts [54,55], which would be essential for space colonies or long voyages to keep the crew fed and healthy as a crop alternative.

## 2. Materials and Methods

### 2.1. Pioneering Focus

Many clinostats are designed to work in 1 or 2 dimensions due to their simplicity [56,57], but a 3-dimensional one is more complex due to the mechanism to make it spin; usually in cases with 2 frames, 2 engines are used and attached to each frame with batteries to maintain the rotation. In this case, as an optimization of a previous work [58,59,60], a single engine should be enough to spin 3 frames in order to get a 3D clinostat sustained by a pulley system, while the electric system to maintain measure or control devices is set through the clinostat axis. Those characteristics were focused on working with terrestrial plants like the tomato cherry [61] and planted in perlite in order to avoid the substrate loss by the rotation, aiming for a space agriculture option [62,63].

**Figure 1:**
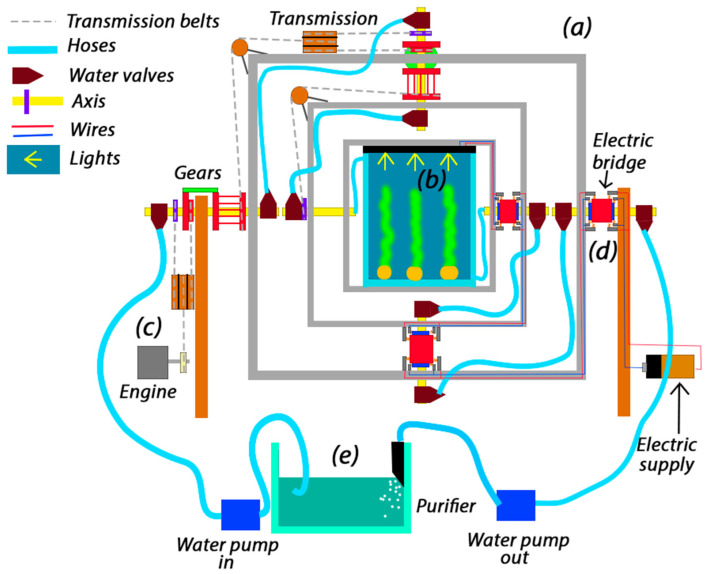
Diagram of the entire system. (a) Clinostat frames. (b) Sample location/water tank. (c) Movement source through the engine and gears. (d) Electric system. (e) Water filtering system and pumps.

For an improvement, the challenge of irrigating the plant without refilling the inner water tank was targeted, developing the system in order to bring liquids to the sample without leaks or refilling needs. Moreover, in order to exploit this feature, a new view was settled with the effects of microgravity in water environments where the filtering and aerating problems are solved to maintain a healthy sample [64], using Figure 1 as the main diagram, being an illustrative image that broadly represents each part of the machine to avoid cluttering the figure with details that will be developed later in each section. Additionally, Figure 1 represents the pioneering conceptual design of an integrated 3D clinostat agriculture system capable of simultaneously combining multi-axis microgravity simulation, automated irrigation, water purification, and electrical transmission within a single compact mechanism. To the best of the authors’ knowledge, this configuration constitutes one of the first biomimetic clinostat-based platforms specifically designed for long-duration terrestrial plant cultivation under simulated microgravity conditions with continuous hydroponic support. Furthermore, this diagram could be modified or adapted for any other project that requires this type of device, given the needs of the researchers.

In contrast with other clinostats, this design is focused to work with any sample which need longer times of uninterrupted experimentation and bigger sizes instead of reducing the scope to only microorganisms and less than 3 axis of microgravity simulation increasing the accuracy of other experiment that require this kind of environment and cares.

### 2.2. The Water Flows

**Figure 2:**
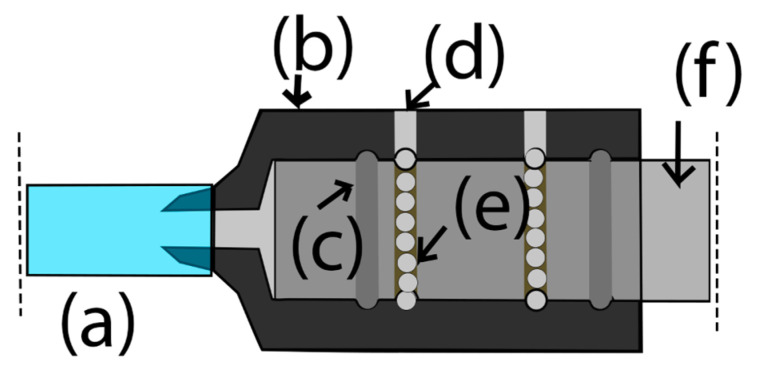
Dotted outline to highlight the swivel joint for the first frame. (a) Water inlet. (b) Joint housing. (c) Seals. (d) Ball bearing inlet. (e) Ball bearings. (f) Hollow shaft of the clinostat.

**Figure 3:**
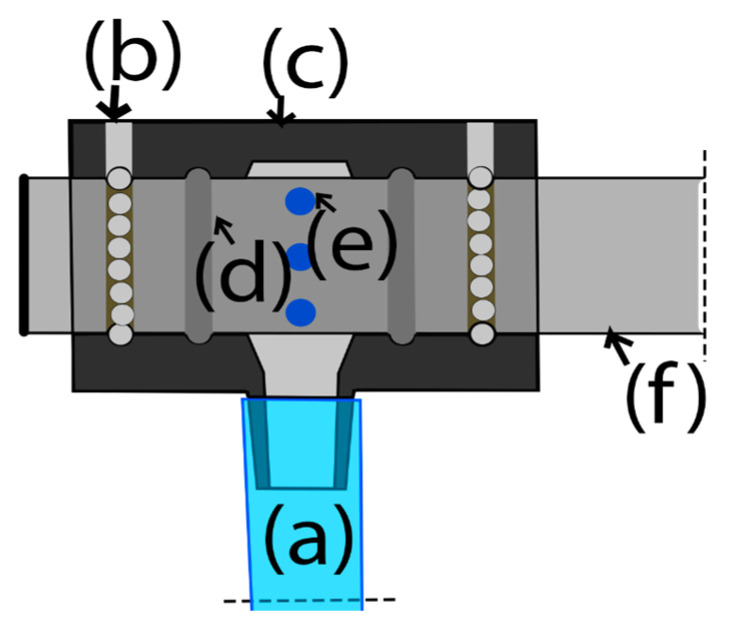
Dotted outline to highlight the swivel joint for inner frames. (a) Water inlet. (b) Ball bearing inlet. (c) Joint housing. (d) Seals. (e) Shaft holes for water flow. (f) Shaft.

Working with water has its own challenges, plus the fact that the moving parts and joints of the machine are prone to leaks [65]. Moreover, the water pressure due to the inertia of rotation could damage the walls of the chamber or even damage the sample [66,67]. For those reasons, a swivel joint mechanism was designed for the exterior (Figure 2) and interior frames (Figure 3).

Due to the possible high pressure needed for flowing water in the machine, 2 pumps are proposed for extracting and injecting the fluid. At the same time, 2 kinds of swivel joints were designed: one for the first entrance, which could receive high pressure, and the other for the inner frames, which could receive less and, to optimize the structure, were designed to be more compact. In contrast, working with living beings increases the maintenance requirements because of nutrients and organic waste [68]. For this reason, a filtering system must be added to avoid a high concentration of toxins due to the rotting of dead leaves or refilling of oxygen if a full ecosystem were to be made [69]. Moreover, some gases could be produced during the experiment, increasing the pressure or microleaks and becoming the filtering system as a water refilling and purifier, as well as a point to take samples or renew nutrients to the environment if needed.

### 2.3. The Automated Aquarium System

**Figure 4:**
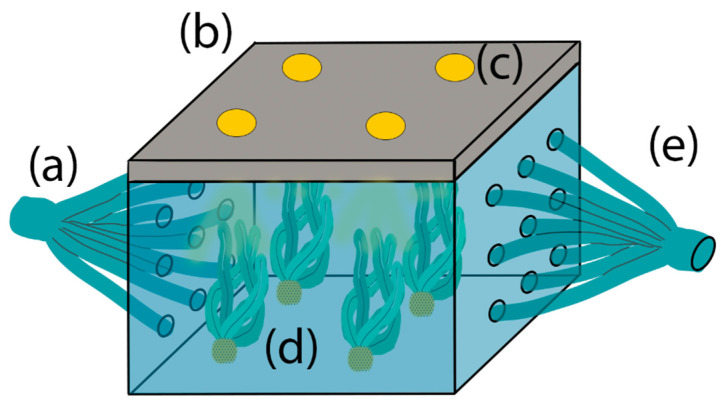
Sample container design. (a) Filling hoses. (b) Lid and surface for adding measuring devices. (c) LED lights. (d) Algae (samples). (e) Drainage hoses.

The project proposal is to design a microgravity simulator in water environments opened to modifications or targets. If a well is proposed to test only small algae, some additions could be made, such as snails, fish, or any other being [70], taking into consideration the dimensions of the chamber and the width of the hoses to avoid sample leaks or even add a mesh for microalgae or small plants, as shown in Figure 4.

Due to the complexity of the chamber shape, the acrylic (polymethyl methacrylate) [71] is one option. It has thermoplastic properties that could help to create roughness in the floor to attach rocks or the substratum for algae supported with mesh. Moreover, it is possible to drill holes in the wall to make the water drain and refill balanced and homogeneously, ensuring a fresh environment. Furthermore, the cover could have a better anchorage to the case with pressure or screws in the walls and, in the same way, fixed to the clinostat frame through screws or mounting clamps.

In the case of the tape, it will be the frame to fix special lights to simulate the day/night hours [72] and a precise wavelength for the specimens. In addition, thermometers [73], pH meters [74], an oximeter [75], cameras, and a food releaser (for animals), among other devices, were used to measure what was needed to study, and all of them were supplied by the electric system. At the same time, this part could be used as a frame for setting rigid guides to fix some parts of the algae in order to ensure a complete spin and avoid the buoyancy effect.

### 2.4. Sample Candidates

It is not feasible for controlled experimentation to test living animals in open water habitats, particularly when the goal is to measure physiological responses to altered-gravity analogs while maintaining constant external variables. Thus, in addition to their nutritional value, the biological candidates chosen for the Phyto-G aqueous clinostat system are also chosen for their suitability for a controlled environment approach in a small chamber. The chamber’s current design allows for artificial illumination and continuous observation, as well as practical surfaces and interfaces for sensors like cameras, dissolved oxygen probes, thermometers, and pH meters [76]. It also permits the use of mesh-like supports or rigid guides to stabilize specimens [77,78] under continuous rotation and lessen buoyancy artifacts.

The primary requirement in this case is that the candidate must tolerate the mechanical limitations of 3D clinorotation while producing interpretable biological signals under strict control of the light regime and water chemistry. Because their thalli are large and can be physically stabilized using mesh or guide structures, edible macroalgae are useful as “first-line” organisms for mechanical validation of the chamber. This reduces uncontrolled drifting and enhances repeatability. However, they continue to produce physiologically significant results, such as growth and color changes associated with pigments [79,80], which can be connected to regulated factors like irradiance and nutrition availability. Due to its widespread use as an edible seaweed and the wealth of research on its nutritional makeup (Table 1) and potential applications, *Ulva lactuca*, also known as sea lettuce, is especially well-suited. Its low lipid content, pertinent carbohydrate and dietary fiber fractions, and variable but significant protein contributions based on processing and cultivation conditions are generally reported [81,82]. Because it enables the system to show that consistent management of light and nutrient supply results in quantifiable variations in biomass and pigment composition across runs, which can be recorded using imaging and simple pigment proxies, this variability enhances the system’s suitability for a controlled environment platform [81,82,83]. *Ulva* is useful because it can be mechanically fixed, is easy to handle in a small volume, and yields morphological and pigment endpoints that are measurable and reflect controlled environmental inputs [81,83].

In addition to *Ulva*, *Pyropia* spp. (nori/laver) provides a macroalgal option with a high dietary relevance, and is often described as a relatively protein-rich seaweed that contains minerals and bioactive substances (Table 2) [84,85]. This is significant for the paper’s overall story: *Pyropia* is frequently cited as an example of a seaweed with advantageous nutritional qualities, and in closed or restricted habitats, the objective is not just growth but also nutritional density per cultivation footprint [84]. It is especially helpful from an experimental perspective because its physiological state reacts to regulated light regimes and stressors; hence, it is conceivable that a chamber with consistent LED schedules could cause detectable changes in growth and pigment-related characteristics. Furthermore, the *Pyropia* literature that emphasizes the impact of biological interactions and environmental context on performance can be used to discuss cultivation stability in closed systems. This supports future work directions where the platform moves from simplified sterile runs to more realistic cultivation ecology [85,86]. All things considered, *Pyropia* is a solid contender due to its high nutritional credibility and continued mechanical tractability for stabilization and repeated monitoring.

Microalgae and cyanobacteria are usually the best options for compact “CEA-like” aquatic farming since they can be cultivated in homogeneous suspension at high density and continually quantified utilizing sensor-friendly outputs, even though macroalgae offer obvious mechanical handling advantages. *Chlorella vulgaris* is therefore suggested as a primary microalgal contender. In addition to its long-standing value as nutrient-rich biomass in controlled cultivation settings (Table 3) [87], *Chlorella* is especially well-suited for a clinostat chamber because its physiological state can be inferred using pigment content and fluorescence proxies (when available), its growth can be measured using optical density and dry biomass, and its system-level function can be monitored using dissolved oxygen and pH dynamics [87]. Most significantly, there is concrete proof that *Chlorella vulgaris* shows detectable physiological plasticity when exposed to altered-gravity analogs based on clinostats. Increased photosynthetic pigments, improved antioxidant-related metrics, and lipidome modification consistent with membrane adaptation can all be linked to simulated reduced gravity circumstances, according to a new npj Microgravity study employing a 3D clinostat [22]. Because of this, *Chlorella* is a strong contender to show that Phyto-G is capable of producing measurable, publishable physiology shifts under changed physical settings in addition to supporting life [22].

Since it combines nutritional significance with remarkably tractable physiological outcomes (Table 4), the cyanobacterium often marketed as Spirulina—often linked to *Arthrospira* platensis in the food and biotechnology literature—is suggested as a second key option [87]. On a dry-weight basis, *Arthrospira* biomass is frequently described as being high in protein. It is also distinguished by unique pigments like phycocyanin, which can be used as a sensitive indicator of changes in cultivation conditions [88]. Practically speaking, this means that the Phyto-G chamber may reasonably link observable outputs like biomass yield, optical density trends, pigment yields, system-level oxygenation, and pH drift to regulated inputs (LED photoperiod, irradiance, nutrition regime, and water chemistry). Furthermore, *Spirulina/Arthrospira* has a wealth of food-grade and biotechnology literature that facilitates quick contextualization of its significance as a contender in limited conditions while offering distinct physiological markers suitable for automated and sensor-based monitoring [88]. As a result, *Arthrospira* is suggested not only due to its edible nature and extensive research, but also because its physiology provides potent quantitative levers that align with Phyto-G’s targeted design elements.

**Figure 5:**
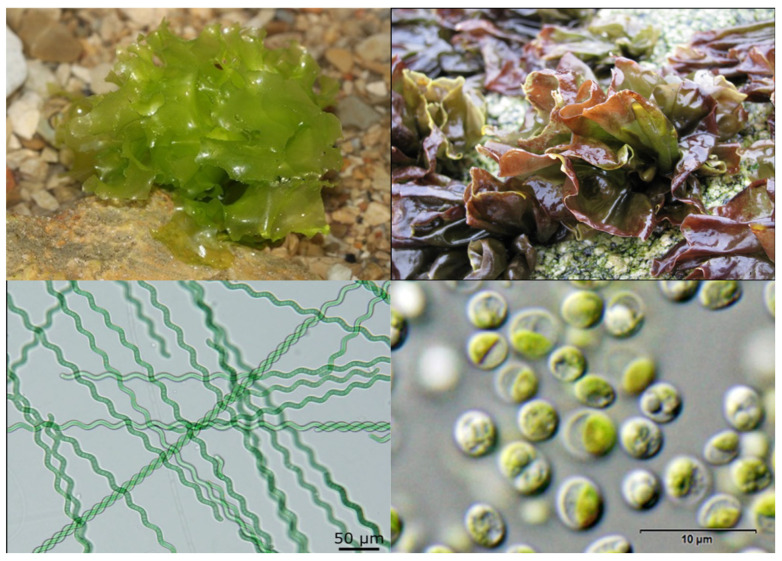
Living beings used for the design of the experiment for illustrative purposes to understand the different morphologies and sizes. Algae: (**Top left**): *Ulva lactuca*, (**top right**): *pyropia*, and micro-algae seen through the microscope. (**bottom left**): spirulina, (**bottom right**): *chlorella*.

When considered collectively, this candidate set is supported by how well it satisfies the mechanical and measurement requirements of the system. In addition to offering clear growth and pigment objectives, the macroalgae *Ulva* and *Pyropia* facilitate chamber handling and stability validation. The scientific justification for the platform’s potential for biological experimentation is strengthened by the fact that the microalgae/cyanobacteria (*Chlorella* and *Arthrospira*) maximize controllability and quantitative monitoring in small volumes and provide direct clinostat-based evidence of altered-gravity-associated physiological plasticity in at least one important organism (*Chlorella*) [87,88] (Figure 5).

## 3. Mechatronic System

### 3.1. Computer-Assisted Mechanical Design and Motion Simulation

This section presents the computational mechatronic design of the 3-axis clinostat prototype based on BEAM-D [89,90], including the mechanical architecture, CAD development, motion study, and structural verification through finite element analysis (FEA). The design is intended as a compact system (≈30 cm envelope) capable of supporting an estimated total payload of ≈30 kg, including water and instrumentation, operating at constant rotation speed (reference outer frame ≈ 10 rpm) and powered by a 24 V supply. The prototype is conceived for lightweight fabrication through reinforced 3D-printable materials and modular interfaces for future experimental adaptations.

#### 3.1.1. Design Requirements

Overall size: ≈30 cm; estimated payload: ≈30 kg (water + devices); operation: constant rotational speed of ≈10 rpm (outer frame); supply: 24 V; manufacturing concept: reinforced 3D printing; design goals: low mass, stiffness, and modularity.

#### 3.1.2. Mechanical Architecture

The proposed mechanical architecture is based on three nested rotational frames (outer, middle, and inner) arranged in a gimbal-like configuration, which together allow multi-axis reorientation of the central sample chamber. In this arrangement, the outer frame is supported by lateral structural columns and connected to the actuation system through a belt–pulley transmission concept, while the middle and inner frames provide the additional rotational degrees of freedom required for clinostat operation. The sample tank is mounted to the innermost frame through a clamp/screw coupling strategy intended to improve stability, modular replacement, and compatibility with future experimental payloads.

**Figure 6:**
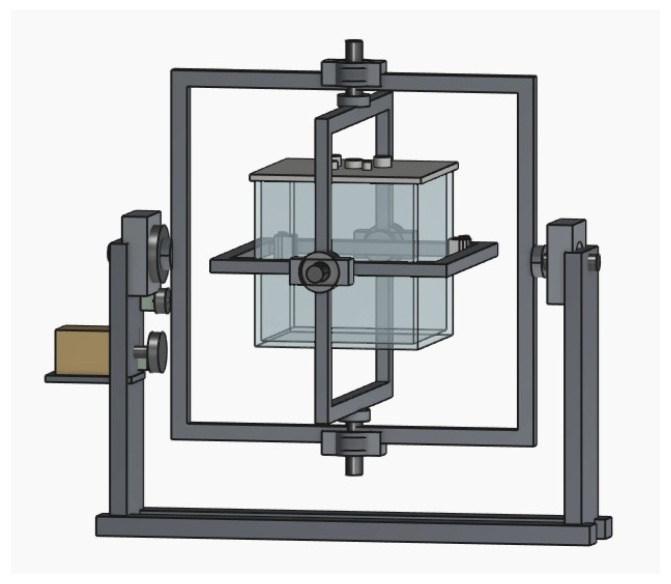
CAD-based mechanical architecture of the proposed 3-axis clinostat.

This mechanical configuration was selected to preserve compactness while still allowing enough internal working volume for the aquatic chamber, auxiliary routing, and shaft integration. The resulting architecture of the proposed clinostat is illustrated in Figure 6.

#### 3.1.3. CAD Model and Assembly

**Figure 7:**
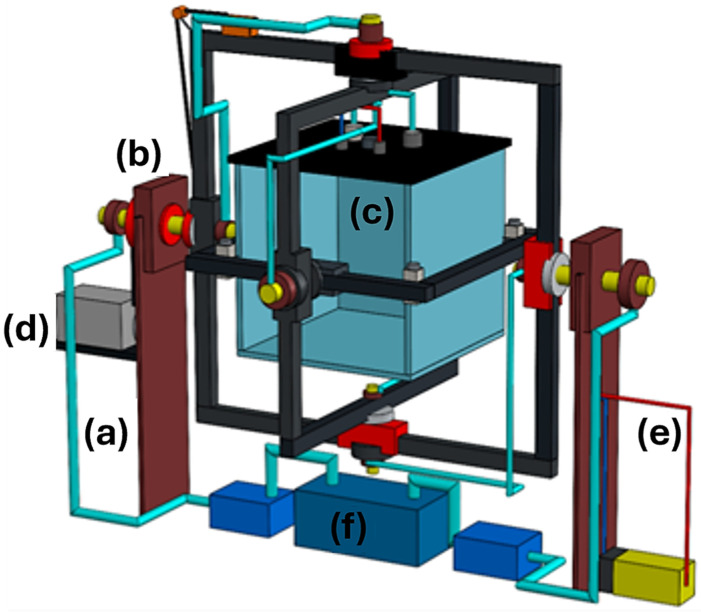
Initial CAD conceptual model of the proposed 3-axis clinostat, preserving the same main components introduced in Figure 1. (a) Including the support columns. (b) Nested rotational frames. (c) Central sample tank. (d) Actuation source. (e)/(f) Auxiliary electrical/fluidic routing elements.

An initial conceptual CAD model of the proposed 3-axis clinostat was developed in SolidWorks 2025 to translate the system-level concept into a realistic mechanical arrangement with clearly identifiable structural and functional components, as shown in Figure 7. The model includes the external support columns, the nested rotational frames, the central sample tank, and the preliminary routing of auxiliary electrical and fluidic elements. At this stage, the objective was to verify the overall geometry of the system and the spatial compatibility between the outer frame, the internal payload volume, and the support/transmission layout. This first complete digital embodiment of the clinostat concept allowed identification of possible interference zones, excessive clearances, and mounting constraints before generating the simplified simulation-oriented assembly. In this way, the CAD model serves as the digital counterpart of the conceptual architecture previously introduced in Figure 1.

**Figure 8:**
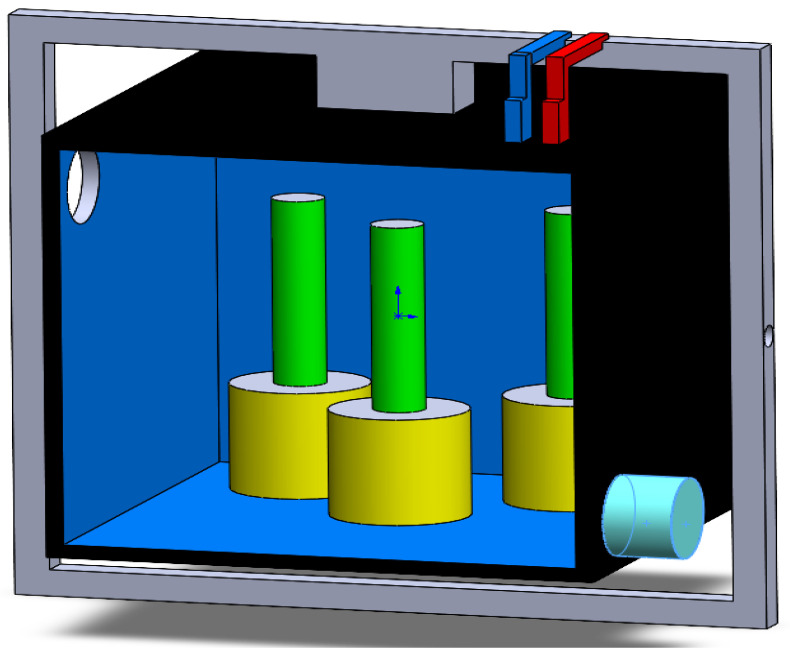
CAD model of the central sample tank and internal payload arrangement used for the aquatic microgravity simulation concept; the green elements represent biological sample columns/culture holders, and the yellow elements correspond to their supporting mounts.

The internal configuration of the central sample tank is detailed in Figure 8. This CAD view was prepared to define the biological test chamber and the arrangement of the main internal payload elements required for the aquatic microgravity simulation concept. In this configuration, the green cylindrical elements represent biological sample columns or culture holders, while the yellow cylindrical bases correspond to their supporting mounts inside the tank. This arrangement was included to verify the available working volume, the spatial distribution of the biological payload, and the compatibility of the tank interior with the innermost rotational frame. In addition, the figure supports evaluation of possible interference between the internal holders, the tank walls, and the service interfaces required for future sensing or fluidic integration. Therefore, Figure 8 complements the overall assembly views by focusing specifically on the sample chamber and its internal support layout.

**Figure 9:**
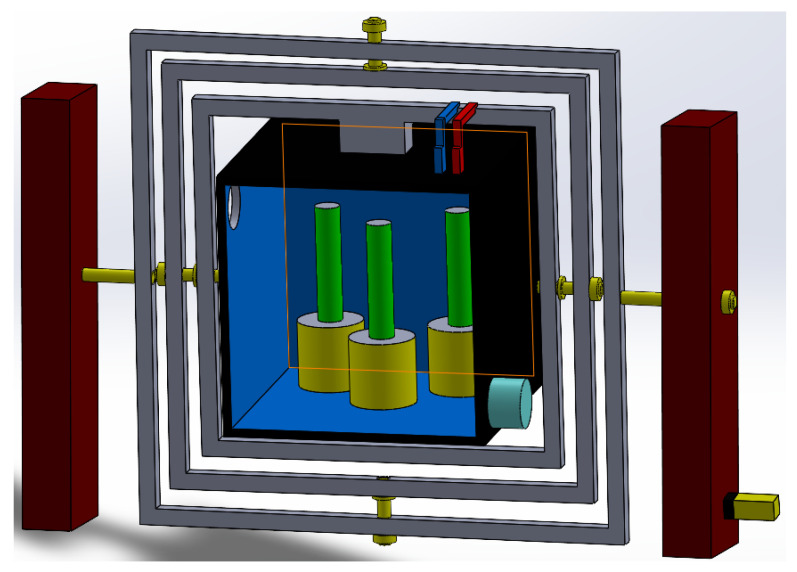
Simplified final CAD assembly prepared for motion and structural simulation, preserving the main rotational frames and shaft interfaces.

After validating the complete conceptual arrangement and the internal payload distribution, a simplified final CAD assembly was prepared for simulation-oriented analysis, as shown in Figure 9. In this version, non-essential external details were removed in order to obtain a cleaner and more computationally manageable model for motion and structural studies. Nevertheless, the main mechanical architecture was preserved, including the external support columns, the three nested rotational frames, the principal shaft interfaces, and the central sample tank. This simplified assembly was used to verify that the reduced simulation model still retained the key geometric relationships and rotational architecture of the original design. Therefore, Figure 9 represents the final CAD configuration adopted as the basis for the subsequent motion study and finite element analysis.

**Figure 10:**
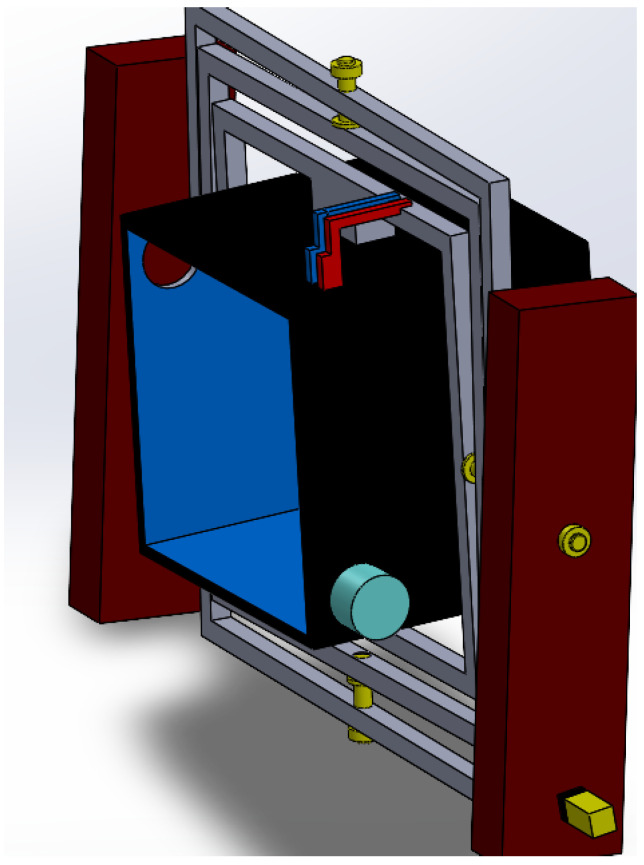
Isometric view of the final 3-axis clinostat assembly used as the reference configuration for the simulation stage.

A final isometric view of the proposed 3-axis clinostat assembly is presented in Figure 10. This representation was included to provide a clear three-dimensional overview of the definitive CAD configuration adopted as the reference model for the simulation stage. Unlike the previous figures, which separately emphasize the conceptual assembly, the internal tank arrangement, and the simplified simulation-oriented model, the isometric view integrates the main structural elements into a single perspective. In this way, the figure allows visual confirmation of the relative positioning of the support columns, the nested rotational frames, the shaft interfaces, and the central sample tank. It also helps illustrate the compactness of the final arrangement and the geometric coherence of the complete mechatronic structure before the motion and FEA analyses were conducted.

#### 3.1.4. Motion Simulation

**Figure 11:**
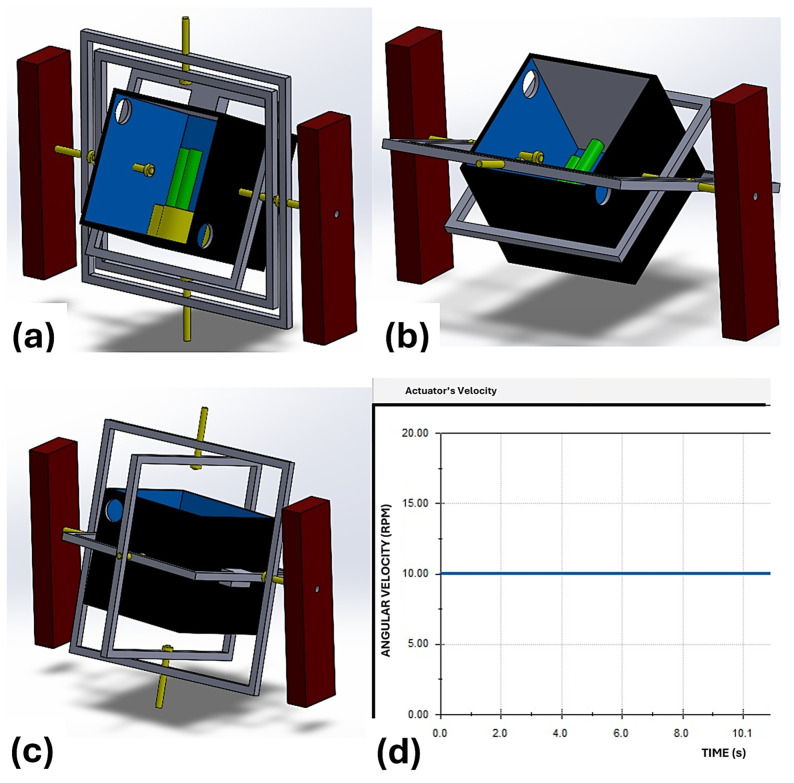
Motion study of the proposed 3-axis clinostat under constant angular velocity (10 rpm): (**a**) initial position; (**b**) intermediate rotational position; (**c**) rotated configuration; (**d**) imposed constant-speed profile used for kinematic validation.

A preliminary motion study was carried out in SolidWorks to verify the kinematic feasibility of the proposed 3-axis clinostat under constant-speed operation. A rotary motor was assigned to the main external axis with a constant speed of 10 rpm, following the design target defined for the outer frame. The simulation confirmed continuous rotational behavior of the assembly and allowed visual inspection of the relative motion between the nested frames and the sample tank. Although the study was performed as a simplified kinematic validation, the obtained results support the feasibility of multi-axis rotation for the proposed prototype. Figure 11 shows representative snapshots of the motion sequence together with the imposed constant-speed profile.

#### 3.1.5. Finite Element Analysis (FEA)

**Figure 12:**
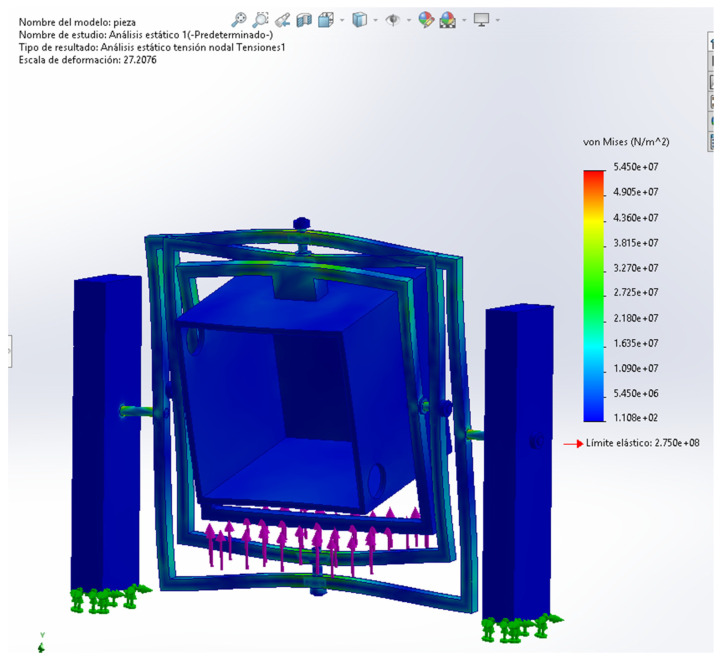
FEA setup and boundary conditions of the simplified full clinostat assembly, showing fixed support conditions and the applied equivalent vertical load.

A preliminary static finite element analysis was carried out in SolidWorks to assess the structural response of the proposed clinostat under an equivalent payload condition. Two simplified validation cases were considered: (i) a component-level analysis of the central sample tank module, and (ii) a full-assembly static analysis of the simplified clinostat structure. In both cases, fixed boundary conditions were applied at the lower support faces, while an equivalent vertical load of 294 N was imposed to represent the estimated payload associated with the tank and instrumentation. A bonded interaction assumption was adopted in the simplified structural model to ensure load transfer between the main solid bodies. The boundary conditions and loading configuration used for the full-assembly case are presented in Figure 12.

**Figure 13:**
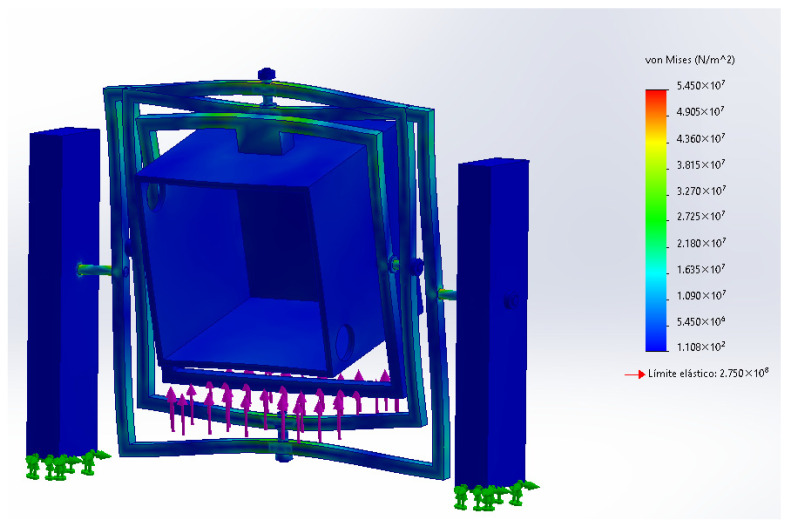
Von Mises stress distribution of the simplified full clinostat assembly under the equivalent static payload.

Once the loading and support conditions were defined, the first result analyzed for the full assembly was the von Mises stress distribution, shown in Figure 13. This plot was used to identify the zones of highest mechanical demand within the simplified clinostat structure and to verify whether the stress levels remained below the selected material threshold in this preliminary assessment. In the evaluated case, the highest stress concentration appeared in the upper structural region of the rotating assembly, with a maximum value of approximately 5.45 × 10^7^ Pa. Although the model was simplified through bonded contacts and a static loading assumption, the result provides a useful first-order estimate of structural feasibility and highlights the main areas that would deserve closer refinement in a future design stage.

**Figure 14:**
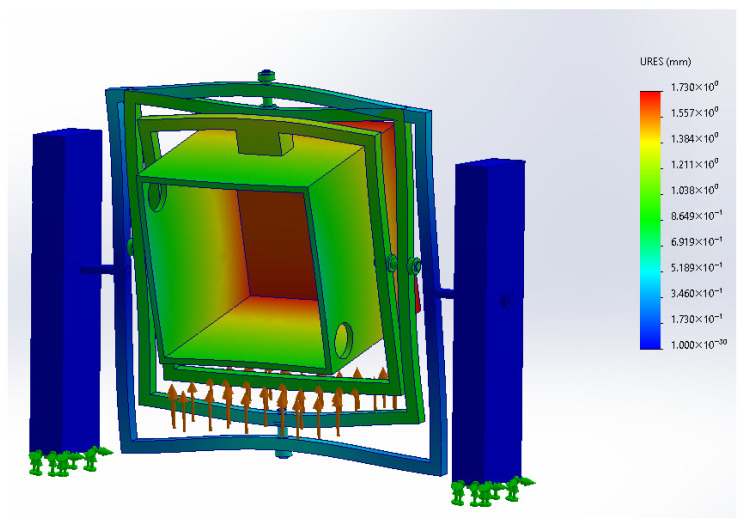
Resultant displacement distribution of the simplified full clinostat assembly under the equivalent static payload displacement distribution under the specified load case.

In addition to the stress distribution, the resultant displacement field was examined in order to estimate the overall structural deformation of the simplified clinostat under the same equivalent load condition. Figure 14 shows the displacement distribution obtained for the full assembly, indicating that the maximum resultant displacement reached approximately 1.73 mm in the most flexible region of the structure. This result suggests that, despite the presence of visible deformation in the amplified simulation view, the global displacement remains within a moderate range for a preliminary conceptual prototype. Together with the stress map, this displacement result supports the mechanical plausibility of the proposed structure under the assumed static loading scenario.

To summarize the preliminary structural verification, the main outcomes of the evaluated static cases are compiled in Table 5. This table includes both the component-level verification of the central sample tank module and the simplified full-assembly case, allowing direct comparison of the applied load condition and the main structural indicators obtained from the FEA stage. In this way, Table 5 serves as a compact summary of the first-order mechanical assessment carried out for the proposed clinostat concept.

### 3.2. Sensor–Actuator Integration

**Figure 15:**
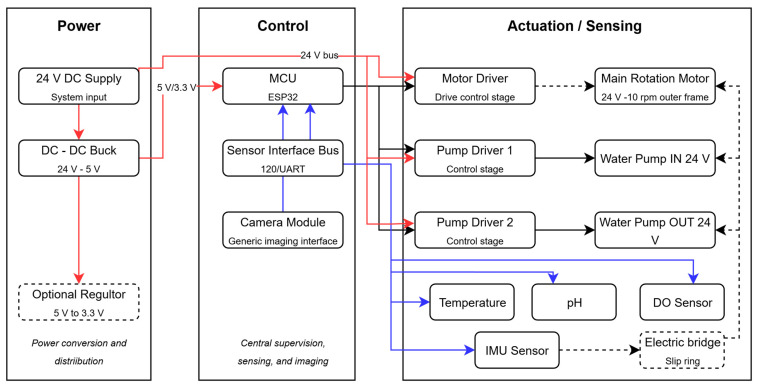
Mechatronic block diagram of the proposed 3-axis clinostat system for aquatic microgravity simulation. The red-colored line is related to the main voltage source, and the blue-colored line is related to the sensor signals acquisition.

This subsection proposes the sensing and actuation elements required for clinostat operation and aquatic environment monitoring. The electrical architecture is based on a 24 V DC main supply with DC–DC conversion to provide regulated rails for control electronics and sensors. The control unit manages constant-speed operation of the main rotary actuator and commands the inlet and outlet pumps to support circulation and filtration. Sensor acquisition is performed through standard interfaces such as I2C, UART, and analog inputs, while a camera module provides visual inspection of the biological samples. The overall interaction between the power source, controller, actuators, pumps, and sensing modules is summarized in the mechatronic block diagram shown in Figure 15.

#### 3.2.1. Actuators

The system includes one main rotary actuator for continuous rotation and two pumps for water inlet/outlet, supporting circulation and filtration. Proposed actuation elements and typical specifications are summarized in Table 6.

#### 3.2.2. Sensors

A minimum sensing suite is proposed: temperature, pH, dissolved oxygen (DO), camera-based observation, and an IMU for motion/vibration monitoring. Proposed sensing elements and interfaces are listed in Table 7.

#### 3.2.3. Electronics Architecture and Schematic

**Figure 16:**
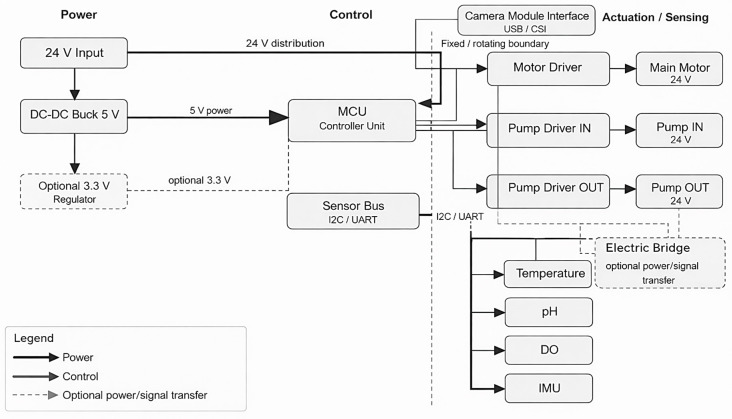
Electronics schematic/wiring overview of the proposed 24 V-based control and sensing architecture.

A simplified wiring overview was prepared to support the future implementation of the proposed system, including the 24 V power distribution stage, DC–DC conversion for logic and sensors, the controller unit, the motor driver, the pump drivers, and the main sensing interfaces. This modular arrangement was conceived to facilitate future integration of additional instruments and to preserve flexibility for different experimental payloads or monitoring needs. The corresponding block-level electronics architecture and wiring overview are presented in Figure 16, which summarizes the main electrical interconnections required for operation of the aquatic clinostat platform.

## 4. Discussion

### 4.1. Extraterrestrial Oceans

With the information obtained thanks to the telescopes, orbiters, probes, and landers, the existence of water in different phases has been found [89], starting with the hypothesis of life outside the Earth [90]. Bringing to the table the possibility of starting longer space missions or permanent colonies. This fact could be taken to start plans of exploiting it for the crew’s biological needs, agriculture, or, even further, pisciculture [91]. However, the gravity conditions could change all the guidelines applied on Earth for them, creating the need to study the microgravity or different gravity effects on living beings [92], as is currently done in the ISS.

### 4.2. The Scope of the Machine

Currently, some experiments were done in the Chinese Space Station with zebrafishes (*Danio rerio*) from eggs until their growth and death (43 days) [93], showing remarkable changes due to microgravity effects. On the other hand, the ISS made the experiment with medaka fish (*Oryzias latipes*), which also affects the new environment. Even if there are some missions that have studied water living beings, there are a lot of species to be tested that could bring an in-depth knowledge of life on Earth. However, the physics limitations of the machine reduce the number of living beings that could be tested. For instance, some plants or algae can be tested due to their poor natural movement and the possibility of being fixed through guides. In contrast, fish with a swim bladder, due to the use of gases to guide their direction, could not be able to be used in the clinostat due to their natural adjustment, plus the impossibility of being fixed to a surface. However, benthic animals like snails, echinoderms, and crustaceans, which mainly move over the seabed or cling to it, could be candidates for the machine due to the “auto-fixing” to the surfaces, being able to be under the simulated microgravity.

### 4.3. The Future of the Agroponics

For this platform, the agroponics future might be characterized as a shift from proof of concept growing to reliable, application-driven testing in regulated aquatic settings. The next step is to formalize a “controlled environment” experimental framework where environmental inputs are programmatically defined, and biological outputs are quantified using standardized endpoints. This is because the Phyto-G chamber incorporates illumination, water renewal, and surfaces appropriate for multi-sensor monitoring (such as pH, dissolved oxygen, temperature, and imaging). This strategy aligns with the ideas of Controlled Environment Agriculture, which emphasizes the precise control of growth variables such as illumination, nutrition delivery, and environmental stability in order to achieve productivity and reproducibility [11]. Although microgravity simulation serves as the driving force behind this effort, the same experimental setup is instantly useful for research and development on Earth and may be subsequently redirected to space-relevant settings with only minor conceptual adjustments.

Establishing high-repeatability growth and physiology baselines for the chosen candidates (*Ulva*, *Pyropia*, *Chlorella*, and *Arthrospira*) under programmable light regimes and specified nutrition inputs is one avenue for future research. This includes tracking oxygen evolution and pH drift as low-cost proxies for photosynthetic performance and culture stability, as well as mapping growth kinetics and productivity under stepped irradiance and photoperiod regimens for microalgae and cyanobacteria. The literature on photobioreactors highlights that maintaining steady productivity in closed systems requires constant observation and management of these factors [12]. Because photosynthetic oxygen accumulation can become a limiting factor, oxygen management in closed reactors is a major engineering challenge and experimental opportunity for *Arthrospira* (*Spirulina* type cultures). This offers a clear pathway for system optimization experiments where aeration strategies, mixing regimes, and gas exchange designs are evaluated alongside productivity [94]. When the study is redirected to space simulations, complementary experiments for Chlorella can combine optical density/biomass endpoints with measurements related to pigment and antioxidants, allowing for a multi-level physiological profile that can subsequently be compared against altered-gravity analog exposures [22].

Utilizing the platform as a small testbed for nutrient recovery and algae-assisted water treatment, two significant terrestrial applications that easily fit a closed aquatic chamber are a second line of inquiry. Microalgae-based systems, such as membrane photobioreactor configurations and controlled cultivation schemes, have been extensively researched for the removal of nutrients (such as phosphate and ammonium) and the reduction in organic load [95,96]. Since the machine already has purification logic, water circulation, and ports that may accommodate periodic sampling, this line of work is particularly complementary to the Phyto-G concept. In practice, experiments can measure biomass yield and nutrient uptake rates at the same time, resulting in a dual output system (biomass generation plus bioremediation) that is appealing for future space-oriented “closed-loop” narratives as well as sustainability applications [97,98].

A third approach involves broadening the scope of potential organisms beyond the original group, emphasizing species that enhance the connection between water farming and the resilience of food systems. In integrated multi-trophic aquaculture settings, macroalgae like *Ulva* are commonly employed as biofilters, converting dissolved nutrients into harvestable biomass. This provides a robust model for experiments measuring nutrient uptake, productivity, and biomass composition under controlled flow and nutrient loading [99,100]. These investigations are directly compatible with the Phyto-G chamber, which allows for the control and replication of nutrient contents, flow patterns, and light regimes. Comparative testing of stability, productivity, and physiological endpoints under similar chamber operation is made possible by the parallel expansion of microalgae candidates to include more robust strains frequently utilized in wastewater or high-density photobioreactor environments [94,97].

Lastly, the platform can be transformed into an automation-focused “biology validation platform” that facilitates repeatable procedures in many labs, such as standardized sensor packages and control scripts for water renewal and lighting. This facilitates terrestrial applications in the short term, including combined cultivation wastewater investigations and nutraceutical biomass optimization (e.g., pigment-targeted culture for *Arthrospira* and antioxidant/pigment objectives in *Chlorella*). Longer-term, conventional controls can be compared to altered-gravity analog exposures using the same automated protocol structure once baseline physiology has been established. This allows for a stepwise transition from terrestrial-controlled cultivation to space-relevant experimental modes while preserving similar measurement endpoints in both scenarios.

## 5. Conclusions and Future Works

The growth of this work makes the 3D clinostat project more complete to address more fields of study and help with the labor-intensive process to contribute to space exploration and human expansion. Moreover, the possibility to simulate environments outside the Earth would contribute to scientists outside the space program or with limited budgets to work in a simulated microgravity. Due to the machine’s versatility, any application can be done in order to adapt the experiment requirements of the scientist, and even more devices or systems could be added to satisfy the needs. If working well, this design is focused on simulating a water environment; it could be changed to a terrain one, by only changing the aquarium with a flowerpot, as in the previous project, as an example, and only using the fluid system for watering.

As mentioned previously, the limitations due to the Earth’s gravity effect as buoyancy are quite difficult to compensate for, making the design of the machine focused on animals whose main tool of orientation is through gases or even algae, with a system to keep on the water surface or near it. However, animals that need a solid surface to survive underwater can be taken for simulation if the aquarium is correctly adapted, even though their movement around the room could have an impact on the microgravity simulated effect, but with many samples, it could be corrected. Step by step, the gap between theory and practice is being reduced. Hypotheses about life outside the Earth could be sustained through simulations in the possible conditions where it could have been developed a long time ago, allowing us to compare what was found and identify any plausible relationship with Earth’s life.

## Figures and Tables

**Table 1:** *Ulva lactuca*: Proximate composition and vitamins/minerals.

Parameter	Reported Value
Moisture (%)	16.9
Ash (%)	11.2
Protein (%)	13.6
Fat (%)	0.19
Carbohydrates (%)	58.1
Dietary fiber (%)	28.4
Vitamin A (IU/100 g)	<0.5
Vitamin B1 (mg/kg)	4.87
Vitamin B2 (mg/kg)	0.86
Sodium (mg/100 g)	364
Calcium (mg/100 g)	1828
Iron (mg/100 g)	14
Potassium (mg/100 g)	467
Phosphorus (%)	0.05

**Table 2:** *Pyropia*/*Porphyra* (laver/nori): Raw laver nutrition (wet vs. dry basis).

Product Type	Species	Carbohydrates (%)	Protein (%)	Lipids (%)	Ash (%)	Moisture (%)	Notes
Raw wet laver	*P. yezoensis*	1.2–2.7	3.0–5.0	0.5	3.6–4.3	89.2–90.5	minerals reported
Raw laver (dry weight)	*P. dentata*	45.7–45.9	36.2–37.7	0.7–1.0	7.1–8.2	8.6–8.8	minerals, amino acids
Raw laver (dry weight)	*P. yezoensis*	51.2–57.9	36.2–39.2	2.3–3.1	3.8–7.3	(not indicated)	minerals, amino acids

**Table 3:** *Chlorella vulgaris*: Nutritional compounds and reported functional relevance.

Chlorella Component	Reported Functional Relevance
Protein	Plant-based protein source for supplementation.
Amino acids	Broad profile; supports tissue/muscle-related nutrition claims.
Polyunsaturated fatty acids	Often discussed in relation to cardiometabolic/anti-inflammatory context.
Polysaccharides	Frequently reported as antioxidant/immunomodulatory bioactives.
Vitamins (B-complex, beta-carotene)	Nutritional fortification; metabolic support.
Minerals (Fe, Mg, Ca)	Micronutrient contribution relevant for constrained diets.
Chlorophyll	Antioxidant and functional ingredient context (also optical endpoint).
Carotenoids (lutein, beta-carotene)	Antioxidant context: “pigment endpoint” for controlled cultivation.
Dietary fiber	Satiety and glycemic modulation context.
Antioxidant pool (chlorophyll/carotenoids/phenolics)	Frequently cited for oxidative-stress mitigation.
Antimicrobial-related compounds (peptides/FA)	Discussed in food/bioprocess applications.

**Table 4:** *Spirulina* (*Limnospira*/*Arthrospira*): Typical dry-weight macronutrient ranges.

Fraction	Typical Range (Dry Weight)	Note
Lipids	5–10%	Reported as a typical lipid fraction in the review.
Carbohydrates	15–20%	Reported as a typical carbohydrate fraction in the review.
Notable fatty acids	GLA (γ-linolenic acid), others	GLA highlighted; varies with culture conditions.

**Table 5:** Summary of preliminary FEA results for the evaluated static cases.

Case	Model	Load Condition	Main Result
Case 1	Central sample tank module	Equivalent vertical load, 294 N	Preliminary component-level verification of tank-support response
Case 2	Simplified full clinostat assembly	Equivalent vertical load, 294 N	Max. von Mises stress ≈ 5.45 × 10^7^ Pa; max. resultant displacement ≈ 1.73 mm; max. equivalent strain ≈ 5.01 × 10^−4^

**Table 6:** Proposed actuation elements for the 3-axis clinostat system (type, proposed model, supply, and key specifications).

Actuator	Type	Proposed Model (Example)	Supply	Key Specs (Typical)	Purpose/Notes
Main rotation motor	DC gearmotor (constant speed)	24 V DC gearmotor (worm/planetary gearbox)	24 V	High torque, continuous duty, low RPM output (via gearbox)	Drives clinostat rotation (~10 rpm outer-frame reference). Final motor selection depends on torque and frame inertia.
Motor driver	High-current DC motor driver	24 V H-bridge/DC motor driver module	24 V logic + power	Current rating ≥ motor stall current; PWM speed control	Enables constant-speed control and direction control if required.
Water pump IN	DC diaphragm/peristaltic pump	24 V DC pump (chemical-resistant)	24 V	Stable flow rate, continuous duty	Water inlet for circulation/renewal; supports filtration loop.
Pump driver 1	MOSFET/relay driver	24 V MOSFET driver module	24 V	On/Off + PWM optional	Controls water pump IN.
Water pump OUT	DC diaphragm/peristaltic pump	24 V DC pump (chemical-resistant)	24 V	Stable flow rate, continuous duty	Water outlet/drain loop; supports purifier circulation.
Pump driver 2	MOSFET/relay driver	24 V MOSFET driver module	24 V	On/Off + PWM optional	Controls water pump OUT.

**Table 7:** Proposed sensing elements for aquatic environment monitoring and clinostat diagnostics (variable, interface, and purpose).

Sensor	Variable Measured	Proposed Model (Example)	Interface	Purpose/Notes
Temperature sensor	Water temperature (°C)	Waterproof digital temperature probe	1-Wire/I2C	Monitoring of aquatic environment stability and experimental repeatability.
pH sensor	pH level	pH probe + signal conditioning module	Analog/UART	Water quality monitoring and algae environment control.
Dissolved Oxygen (DO) sensor	DO concentration (mg/L)	DO probe + interface module	Analog/UART/I2C	Ensures viable oxygen levels for aquatic samples; key variable for long observation times.
IMU sensor	Acceleration & angular rate	6/9-DOF IMU module	I2C/SPI	Motion/vibration monitoring; supports validation/diagnostics during rotation.
Camera module	Visual observation	Camera module (generic)	USB/CSI	Visual inspection, sample tracking, documentation of changes during experiment.

## Data Availability

The original contributions presented in the study are included in the article. Further inquiries can be directed to the corresponding author.
